# Engineered mesenchymal stem cell-derived exosomes: A revolutionary approach to unlocking liver disease treatment

**DOI:** 10.1016/j.bbrep.2025.102313

**Published:** 2025-10-31

**Authors:** Amir Hossein Kheirkhah, Mohsen Sheykhhasan, Faezeh Hosseinzadeh, Leyla Fath-Bayati

**Affiliations:** a Student Research Committee, Qom University of Medical Sciences, Qom, Iran; b Cellular and Molecular Research Center, Qom University of Medical Sciences, Qom, Iran; c Department of Tissue Engineering and Applied Cell Sciences, Faculty of Medicine, Qom University of Medical Sciences, Qom, Iran; d Clinical Trial Center, Qom University of Medical Sciences, Qom, Iran

**Keywords:** Exosome, Mesenchymal stem cells, Liver, Regeneration

## Abstract

Exosomes, a specific type of extracellular vesicle with diameters ranging from 30 to 150 nm, play a vital role in coordinating various physiological and pathological processes through direct cell-to-cell communication. These vesicles transport a diverse range of molecules, including proteins, mRNAs, miRNAs, and lipids, that originate from parent cells. Exosomes are released by nearly all cell types, including mesenchymal stem cells (MSCs), and possess significant potential due to their regenerative and immunomodulatory properties. In the context of liver disease treatment, these capabilities offer hope. Despite their limited lifespan in the bloodstream and suboptimal targeting efficiency, researchers are exploring the potential of modifying MSC-derived exosomes through genetic or chemical means. This article highlights the promising characteristics and applications of MSC-derived exosomes, presenting new perspectives on the latest advancements in exosome engineering for liver regeneration and therapy.

## Highlights

• Engineered MSC-derived exosomes offer a novel strategy for liver disease therapy.
• MSC exosomes exhibit strong regenerative, anti-fibrotic, and immunomodulatory effects in the liver.
• Genetic and chemical modification enhances the targeting efficiency of exosome therapy.
• Exosome-mediated delivery of miRNAs supports liver regeneration and reduces inflammation.
• Preclinical studies confirm that MSC exosomes reduce acute liver injury and fibrosis.

## Graphical abstract

**Figure:**
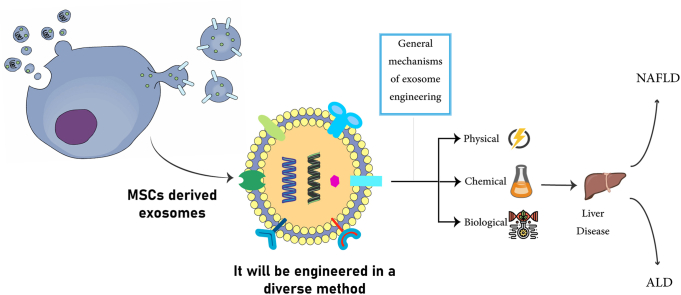

## 1 Introduction

The treatment of liver disease is challenging due to its complexity and numerous complications. Additionally, late treatment can have a profound impact on a patient's overall well-being and standard of living [1]. Extraneous variables such as toxic chemicals, viruses, and a diet heavy in fat can lead to early liver diseases that, if not diagnosed and treated in time, may progress to end-stage hepatic disease [[2], [3], [4]]. Liver diseases are separated into categories of acute and chronic liver illnesses. Acute liver failure is a dangerous condition with a frequency of fewer than 10 reported cases per million individuals annually in the developed world that, although uncommon, can be fatal. Acute liver failure is a pathological state that may manifest suddenly and pose a severe threat to life. This happens when the liver cells become damaged or die. Several factors can cause acute liver failure, such as viral hepatitis (hepatitis A and C), alcohol abuse, nonalcoholic fatty liver disease, autoimmune disorders, infectious diseases, medication side effects, and genetic disorders. Some of the symptoms that may indicate acute liver failure include jaundice (yellowing of the skin and eyes), nausea, vomiting, and abdominal pain. In the US and much of Western Europe, the most frequent cause of abrupt liver failure is drug-induced liver injury [5,6]. The two most common types of chronic liver illnesses with modest clinical signs are metabolic-associated fatty liver disease (MAFLD) and alcohol-associated liver disease (ALD). However, acute episodes caused by increasing alcohol use might exacerbate the pathophysiology of ALD and cause signs of the acute clinical condition known as alcoholic hepatitis (AH). ALD and MAFLD or nonalcoholic steatohepatitis (NASH) can both cause cirrhosis and fibrosis and increase the likelihood of developing hepatocellular cancer. Most chronic liver diseases activate a proinflammatory response in the liver and circulation, driven by innate immune cells [7,8]. MAFLD is closely associated with dietary patterns, and increasing evidence indicates that nutritional interventions, such as Mediterranean-style diets rich in omega-3 fatty acids and antioxidants, while low in refined sugars, can significantly help prevent the onset of the disease and influence its progression [9,10].

**Fig. 1:**
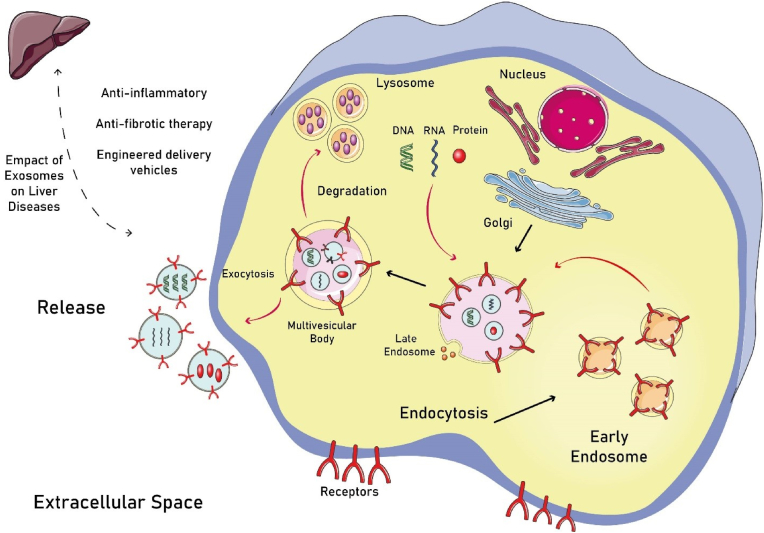
The biogenesis process of exosome release from cells and the beneficial effects of exosomes derived from mesenchymal stem cells on liver disease. The biogenesis of exosomes involves a complex process in which cells produce and release small extracellular vesicles that are essential for intercellular communication. These exosomes originate from the inward budding of the plasma membrane, leading to the formation of early endosomes, which then mature into multivesicular bodies (MVBs). Upon fusion of MVBs with the plasma membrane, exosomes are released into the extracellular space. These exosomes are rich in various bioactive molecules, including proteins, lipids, mRNA, and microRNA, which can modulate cellular functions in recipient cells. Research has demonstrated that MSC-derived exosomes can contribute to liver regeneration, reduce inflammation, and promote cellular repair and survival in hepatic tissues. By transferring beneficial molecules to damaged liver cells, these exosomes help mitigate the progression of liver diseases, such as fibrosis, steatosis, and cirrhosis, making them a promising avenue for therapeutic interventions.

Liver tissue damage, including extensive liver cell necrosis in end-stage hepatic disease, impairs the organ's ability to repair itself. Under these conditions, the primary treatment option is liver transplantation; however, despite its significant benefits, several issues remain. These challenges include a shortage of suitable donors, high costs, numerous risks such as immune reactions against the transplanted tissue, and a difficult recovery period. As a result, developing new and effective therapies for liver disease has become a widely researched topic [9,10]. Three groups of extracellular vesicles are distinguished by their origin, size, and how they are released from cells: microvesicles, exosomes, and apoptotic bodies. Exosomes, which range from 30 to 150 nm in size, have received considerable attention due to their unique properties [11]. They can target specific cells through surface and adhesion proteins, transferring information and promoting various signaling pathways. Because they contain proteins, RNA, and other components from their parent cells, exosomes can exert therapeutic effects by influencing the function of target cells [12]. Previous studies have shown that noncoding RNAs secreted by exosomes, including small noncoding RNAs (such as microRNAs (miRNAs)), long noncoding RNAs (lncRNAs), and circular RNAs (circRNAs), play key roles in their hepato-protective effects [13]. These noncoding RNAs regulate gene expression, genomic imprinting, and nuclear transport at the post-transcriptional level [[14], [15], [16]]. Recent research indicates that exosomes derived from mesenchymal stem cells (MSC-Exos) positively impact a variety of liver diseases, including drug-induced acute liver injury, liver fibrosis, and hepatocellular carcinoma (see Fig. 1) [[17], [18], [19], [20]]. Compared to MSCs, exosomes offer advantages such as improved safety, reduced immunogenicity, targeted therapy, and easier storage and delivery [20]. Additionally, bioengineering exosomes or their parent cells is a viable approach. Therefore, MSC-derived exosomes show potential as an optimal therapeutic option for liver diseases. Hence, this review examines the therapeutic potential, engineering strategies, and translational perspectives of MSC-Exos for treating liver diseases, synthesizing current experimental evidence and outlining technological and clinical pathways for future application.

## 2 Challenges posed by mesenchymal stem cells in liver regeneration

MSCs can be acquired from different locations in the body, including the bone marrow, placenta, umbilical cord, adipose tissue, and dental pulp [11]. These cells are widely utilized to treat a variety of illnesses, such as cardiovascular, kidney, liver, and neurological diseases, due to their exceptional capacity for self-renewal, multidirectional differentiation potential, ease of isolation and expansion, and low likelihood of rejection by the immune system, thanks to their immunoregulatory effects [12]. Numerous studies indicate that MSCs, when expanded *in vitro* under specific conditions or transplanted *in vivo* into living organisms, often exhibit characteristics of liver cells. Multiple research groups have demonstrated that these cells express several proteins, including albumin, alpha-1-antitrypsin, alpha-fetoprotein, fibrinogen, glycogen, and additional advanced hepatic markers, such as drug-metabolizing genes like CYP3A4, as seen in liver cells [13,14]. The ability of MSCs to develop into cells with an advanced hepatic phenotype upon transplantation may determine their suitability as a source to obtain hepatocyte cells. Despite the limited evidence that MSCs from various sources can develop into mature hepatocytes *in vitro* or *in vivo*, there is growing evidence that these cells may enhance liver function when injected into patients with cirrhosis [15]. In addition, multiple functional roles of MSCs have been reported in numerous studies, supporting the potential of these cells in liver restoration through paracrine, cell-cell contact, and homing mechanisms [[16], [17], [18], [19], [20], [21]]. First, it is thought that MSCs migrate to injury sites and differentiate into target cells to repair damage; however, recent research has demonstrated that their ability to differentiate at the location of injury is inconsistent and that their engraftment success rate is low [22,23]. Instead, extensive research has demonstrated that secretions from MSCs, including growth factors, cytokines, and chemokines, released through paracrine action and exosomes, exhibit therapeutic effects that are similar to or even greater than those of MSCs themselves. These secretions have anti-inflammatory, antifibrotic, and proangiogenic effects at the injury site [20,[24], [25], [26], [27]]. The following are some potential benefits of MSCs: 1. Promoting regeneration: MSCs produce molecules that stimulate the growth and division of existing liver cells (hepatocytes) after injury. This can accelerate the liver's natural regeneration process. 2. Immunomodulatory effects: MSCs can modulate the liver's inflammatory response, thereby mitigating damage. This creates a more favorable environment for healing. 3. Antifibrotic properties: Liver injury can lead to fibrosis, a process of scarring. MSCs may help reduce fibrosis by inhibiting scar formation [[28], [29], [30]]. Although preclinical data are encouraging, several challenges must be addressed to translate MSC therapy into a robust clinical approach. These challenges include limited differentiation capacity, delivery strategies, and safety and optimization protocols [31,32].

## 3 Exosomes and their characteristics

Extracellular vesicles are nanosized vesicles that are released from different cells and separated into three groups: microvesicles (MVs) (20–1000 nm) derived from the cytoplasmic membrane; apoptotic vesicles (50–5000 nm) produced during cell apoptosis; and exosomes (30–150 nm) released by exocytic fusion of endosomes with the cell surface [33]. Many human cells, such as MSCs, B cells, macrophages, T cells, and dendritic cells, can secrete exosomes in body fluids such as tears, breast milk, saliva, blood, and urine, or into cell culture supernatants [33]. Exosomes, often referred to as small extracellular vesicles, exhibit considerable size variability across studies. This variability is largely determined by factors such as the cell type or biological source, physiological conditions, and the methods employed for isolation and measurement. For instance, some cell populations may generate vesicles that surpass the traditionally recognized size range, underscoring the need to account for exosome origin in research [[34], [35], [36]]. Exosome formation is thought to occur in three stages, starting with endocytic vesicles formed from cell membrane invagination, then the formation of multivesicular bodies (MVBs) from endocytic vesicles, and finally, exosome release through fusion with the cell membrane. The endosomal sorting complexes required for transport (ESCRT) regulate exosomes, as well as quadruple transmembrane proteins, ceramide, and enzymes from the Rab GTPase family [[37], [38], [39]]. Exosomes from various origins often have distinct contents, making them valuable biomarkers for specific tissue-derived exosomes in the clinical treatment of multiple diseases [40]. Exosomes comprise a mixture of substances, including proteins, lipids, DNA, and RNA, of which miRNAs are particularly noteworthy due to their role in cellular processes such as adhesion, membrane fusion, metabolism, and signaling [[41], [42], [43], [44]]. Exosomes exhibit minimal toxicity and immunogenicity and lack the capacity to induce malignancies, making them ideal agents for drug delivery, particularly antitumor drugs [40]. Like other exosomes, MSC-Exos contain proteins such as membrane-associated proteins (MHC-I, MHC-II, CD63, CD81, and CD9), heat shock proteins (Hsp8, Hsp90, and Hsp70), and MVB proteins (syntenin, TSG101, and ALIX) [45,46]. In addition, MSC-Exos contain unique proteins, including CD29, CD44, and CD73 [47]. However, exosomes may exhibit marked heterogeneity in surface and cargo proteins. While tetraspanins such as CD63, CD81, and CD9, along with cytosolic proteins including Alix and TSG101, are widely accepted as general exosome markers, their abundance is not uniform across all exosome populations. Variations in marker expression have been observed between exosomes derived from different tissues, developmental stages, or culture conditions [34,48,49]. Furthermore, MSC-Exos include substances specific to their biological sources, such as particular non-coding RNAs (ncRNAs), saturated fatty acids, and lipid rafts, all of which regulate the impact of MSC-Exos on target cells [47].

### 3.1 Experimental evidence and characteristics of exosomes derived from mesenchymal stem cells

The role of MSC-derived exosomes is not yet fully understood; however, they appear to facilitate communication between cells, similar to exosomes from other cell types. In their interactions with multiple cells, they elicit responses and maintain the tissue environment [50]. MSC-Exos contain a complex mixture of genetic material, proteins, and lipids, including over 4850 unique gene products and 4150 microRNAs [51,52]. The components discovered in MSC-derived exosomes are involved in various processes, including communication, immune system regulation, energy production, tissue regeneration and repair, and metabolic pathways. This diversity of functions highlights the potential of MSC-derived exosomes to engage with various cell types, causing diverse responses [53]. MSC-exosomes and MVs have therapeutic effects on various injury models, including those affecting the heart, nervous system, lungs, and acute kidney injury. MSC-Exos and MVs have been shown to enhance renal function and mitigate kidney injury in different models of kidney injury [[54], [55], [56], [57]]. They can also alleviate inflammation and promote cell proliferation in response to kidney injury. In myocardial injury, the administration of MSC exosomes before treatment has been demonstrated to decrease the magnitude of the infarct and improve heart function [[58], [59], [60], [61]]. Certain studies have revealed that exosomes derived from MSCs possess protective qualities against heart injury by working through various mechanisms, including preventing cell death, promoting heart tissue regeneration, reducing inflammation, facilitating the formation of new blood vessels, and counteracting changes in blood vessels [62,63]. MSCs have gained popularity for treating tumors, as they can migrate from their origin sites to the surrounding tumor area [64,65]. Studies have yielded mixed results regarding whether MSCs promote or inhibit tumor growth, and the impact of MSCs on tumor cells remains unclear. Paracrine factors secreted by MSCs and delivered via EVs can affect tumor progression [66]. In some studies, exosomes derived from MSCs of multiple myeloma patients have been found to promote tumor growth in mice. In contrast, exosomes from human MSCs have been found to impede the proliferation and viability of human tumor cell lines [[67], [68], [69], [70]]. In a separate study, researchers found that exosomes derived from MSCs inhibited the progression of breast cancer and blood vessel formation by reducing the levels of a growth factor [71]. The influence of exosomes generated from mesenchymal stem cells on tumor proliferation may be inconsistent. A study revealed that exosomes from menstrual stem cells prevented the growth of prostate tumors by decreasing the production of factors that promoted tumor growth in a mouse model of prostate cancer [72]. The varying effects of MSC-derived exosomes on tumor growth may result from differences between MSCs and the exosomes they secrete, particularly in terms of specific cell molecules, tumor type, and method and timing of exosome administration [73]. The use of MSC-derived exosomes in cancer treatment must be approached cautiously, as their impact on tumor growth is not yet fully understood. A comprehensive understanding of the control mechanisms of MSC-derived exosomes is crucial for determining how they contribute to the spread of cancer and for directing the development of effective therapeutic agents through targeted modification [53]. Exosomes derived from MSCs not only affect tumor development but also impact tumor chemosensitivity. In a subcutaneous xenograft tumor model in BALB/c nu/nu mice, human umbilical cord MSC-derived exosomes increased the tolerance of gastric cancer cells to 5-fluorouracil by preventing 5-fluorouracil-triggered apoptosis and increasing the expression of multidrug resistance-related proteins [74]. Another study revealed that exosomes from bone marrow-derived MSCs (BM-MSCs) containing anti-miR-9 reversed the resistance of glioblastoma multiforme cells to chemotherapy drugs, such as temozolomide, by altering the expression of the multidrug transporter P-glycoprotein [75].

### 3.2 Obstacles in the application of exosomes derived from mesenchymal stem cells

**Fig. 2:**
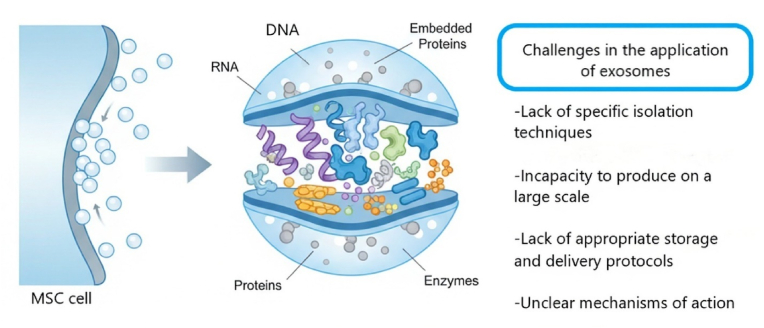
Several significant hurdles exist in the application of exosomes for therapeutic purposes. Major obstacles include a lack of specific isolation techniques and difficulty in large-scale production. Additionally, there are no standardized storage protocols for exosomes. The mechanisms of action and optimal delivery methods remain unclear. Ensuring clinical-grade sterility of exosomes is also a critical requirement for safe application.

MSC-derived exosomes possess significant potential for regenerative medicine; however, their application faces substantial challenges (see Fig. 2). One of the most critical obstacles to the clinical use of exosomes is the lack of a specific isolation technique. Various techniques, including precipitation, ultracentrifugation, ultrafiltration, flushing separation, microfluidic isolation, antibody affinity capture, and mass spectrometry, have been developed to extract exosomes from biological fluids [76,77]. The most commonly used method is differential ultracentrifugation, often paired with a density gradient to enhance purity; however, it can be time-consuming, labor-intensive, and may lead to vesicle deformation or aggregation [78]. While it offers high purity and preserves vesicle integrity, it typically results in relatively low concentrations, making it less suitable for large-scale applications. On the other hand, alternative methods are rapid and scalable but may lead to membrane clogging and shearing of the vesicles [79]. Furthermore, Polymer-based precipitation kits are effective for recovering exosomes with minimal equipment, but they can also co-precipitate contaminants such as protein aggregates and lipoproteins. Immunoaffinity capture, which utilizes antibodies targeting specific exosomal surface proteins like CD63, CD81, and CD9, results in highly pure vesicles; however, this method is costly and has a limited capacity. Recently, asymmetric-flow field-flow fractionation (AF4) has emerged as a technique for precise size-based separation, though it requires specialized instrumentation [78,80,81]. Totally, these procedures are arduous, laborious, and costly, lacking established norms [82]. The absence of distinctive surface markers and the presence of other extracellular vesicle types, such as microvesicles, sometimes result in the simultaneous isolation and contamination of collected exosomes [83,84]. Conventional approaches, such as ultracentrifugation, can alter morphology and functionality. The isolation of exosomes by high-speed pelleting can result in mechanical damage, membrane distortion, protein aggregation, lipoprotein contamination, and reduced purity [85]. Storage protocols have a significant impact on the utilization of exosomes in regenerative medicine. The changes are more noticeable at four and −20 °C than at −80 °C. For example, the levels of CD63 and HSP70 decrease at 4 °C over ten days. In addition, exosomal cargo loss is greater at room temperature. Freezing/thawing cycles lead to exosome aggregation. Therefore, understanding cryopreservation mechanisms is crucial for maintaining exosomal integrity and function [[86], [87], [88]]. The sterility of exosome therapy is essential because the exosome fraction can contain virions, viral products, toxins, and bacteria-associated vesicles. Microbes and fungi are typically scarce in the exosome fraction due to filtration [89]. Certain retroviruses can exploit the exosome biogenesis process to spread within the body. The presence of virus-related genetic material and proteins increases the risk of infection and can change the action of parental cells. Consequently, it is important to carefully screen parent cells for dormant viral infections before large-scale production of exosomes for *in vitro* systems [90]. The extensive use of exosomes for therapeutic purposes through systemic administration may increase the risk of thrombosis, which is related to the concentration of exosomes. The presence of tissue factor and phosphatidylserine is precisely linked to the risk of thrombosis, with large-sized exosomes containing greater amounts of pro-thrombotic factors than small-sized exosomes. Compared with exosomes purified from parent cells *in vitro*, those isolated from biofluids may have a greater risk of thrombosis because of tissue factors and other procoagulant factors [88,91].

## 4 The biomedical applications of MSC-derived exosomes

Exosomes derived from MSCs have multiple biomedical applications due to their natural bioactive cargo, biocompatibility, and ability to cross biological barriers. One of the primary areas that utilizes them is regenerative medicine. MSC-Exos support tissue repair by transferring miRNAs, proteins, and growth factors that promote cell proliferation, angiogenesis, and extracellular matrix remodeling. Preclinical studies demonstrate effectiveness in regenerating damaged liver, myocardium, neural tissue, and skeletal muscle [92,93]. Another application of MSC-Exos is in drug and gene delivery. They use their inherent targeting properties and stability to transport therapeutic molecules, including small drugs, siRNAs, miRNAs, plasmids, and CRISPR/Cas9 components, directly to specific cells. This process bypasses endosomal degradation and significantly improves delivery efficiency [94,95]. MSC-Exos are also effective for immunomodulation, as they influence immune cell differentiation and function. They bias macrophage polarization, suppress the release of pro-inflammatory cytokines, and reduce autoimmune responses, which are vital for graft survival and treating inflammatory diseases [96,97]. Additionally, MSC-Exos display potent anti-fibrotic and anti-cancer effects. By delivering anti-fibrotic miRNAs, such as miR-122 and miR-148a, or tumor-suppressive molecules like miR-451a and miR-338-3p, MSC-Exos effectively inhibit hepatic stellate cell activation, decrease collagen buildup, slow tumor growth, and increase chemosensitivity in hepatocellular carcinoma models [[98], [99], [100]]. Ultimately, the most significant application of exosomes, especially MSC-Exos, is in diagnostics and biomarker development, where their cargo reflects the physiological or pathological state of their parent cells, making them promising candidates for non-invasive biomarkers in early disease detection, prognosis, and treatment monitoring [101,102].

## 5 The therapeutic efficacy of exosomes originating from mesenchymal stem cells in treating liver disease

The self-renewal capacity and multiple abilities of MSCs make them desirable options for managing hepatic disorders. Recent research has shown that exosomes derived from MSCs are as effective as progenitor stem cells in repairing various injuries to different organs, primarily by transferring their contents to recipient cells and inducing functional and visual changes [92,93]. Recent preclinical investigations have documented the therapeutic and regenerative effects of MSC-derived exosomes in liver diseases; some of these effects are summarized in Table 1 [23,[94], [95], [96]].

**Table 1:** Several critical investigations have been conducted regarding the therapeutic function of MSC-derived exosomes in hepatic disorders.

Cell type	Disease	Type of study	Therapeutic mediator	The resulting implications	References
AMSC	Primary hepatic carcinoma	*In vitro*	miR-122	Improve response to chemotherapy	(97)
	Liver fibrosis	*In vivo* preclinical	miR-122	Repair liver fibrosis	(97)
	Acute liver injury and liver failure	*In vivo* preclinical	miR-17	Ameliorate liver dysfunction caused by LPS/GalN	(98)
Amnion-MSC	Liver fibrosis	*In vivo* preclinical	Not mentioned	Decrease liver fibrosis and activation of HSCs and KCs	(99)
HUC-MSC	Liver fibrosis	*In vivo* preclinical	Not mentioned	Ameliorate hepatic fibrosis brought on by CCl4	(100)
BM-MSC	Liver fibrosis	*In vivo* preclinical	Not mentioned	Suppress HSC activation	(101)
	Acute liver injury and liver failure	*In vivo* preclinical	Not mentioned	Regulating inflammatory reactions can lessen liver damage	(102)
CP-MSC	Acute liver injury and liver failure	*In vivo* preclinical	miR-125b	Alleviate liver fibrosis	(95)
SHED	Acute Liver Injury and Liver Fibrosis	*In vivo* preclinical	More investigation is needed	Liver Regeneration, Anti-fibrotic activity, and Anti-inflammatory Effects	(103)

**Abbreviations:** AMSCs, adipose-derived mesenchymal stem cells; KCs, Kupffer cells; HSCs, hepatic stem cells; HUC-MSCs, human umbilical cord mesenchymal stem cells; BM-MSCs, bone marrow mesenchymal stem cells; CP-MSCs, chorionic plate-derived mesenchymal stem cells; SHEDs, stem cells derived from human exfoliated deciduous teeth.

### 5.1 Using exosomes delivered from MSCs to treat acute liver failure

Thus far, few studies have investigated the use of exosomes derived from MSCs as a treatment option for acute liver injury [104]. Exosomes derived from MSCs strongly reduce the systemic inflammatory reaction in animal models of acute liver failure (ALF) by impairing the IL-6-mediated signaling axis and downregulating the NLRP3 pathway [105,106]. Oxidative stress plays a significant role in the progression of liver injury, especially in conditions like MAFLD, viral hepatitis, and damage caused by toxic substances. Research has shown that MSC-Exos can reduce reactive oxygen species (ROS) by delivering antioxidant enzymes, such as glutathione peroxidase 1 (GPX1) and superoxide dismutase, as well as regulatory microRNAs like miR-146a/Src. These components work together to enhance Nrf2-mediated antioxidant pathways [[107], [108], [109]]. Recently, exosomes enriched with GPX1 derived from UC-MSCs have been shown to alleviate oxidative stress and apoptosis in hepatocytes, resulting in a hepatoprotective effect in ALF rodent models [110]. Tan and colleagues discovered that exosomes derived from HuES9. E1 MSCs derived from human embryonic stem cells (hESCs) protect against liver injury caused by acetaminophen or H2O2 *in vitro* and against CCl4-induced injury in C57BL/6 mice. This is primarily due to the strengthening effect of MSC-derived exosomes on liver cell proliferation, which is indicated by increased levels of proliferating cell nuclear antigen and improved cell viability. The overall survival rate is related to the overexpression of genes involved in liver regeneration, resulting in increased levels of proliferation proteins, antiapoptotic genes, and the STAT3 signaling molecule. However, the therapeutic efficacy appears to be unrelated to the modulation of oxidative stress in liver damage [104]. In a groundbreaking study, Shao et al. reported that exosomes release miR–455–3p and effectively initiate PI3K signaling, leading to sustained hepatocyte proliferation. This finding is promising, especially considering that miR–455–3p–enriched exosomes have a remarkable ability to suppress macrophage activation, reduce regional hepatic damage, and downregulate the synthesis of proinflammatory cytokines *in vivo*, suggesting a potential avenue for liver injury treatment and regenerative medicine [105,111]. miR-455–3p can also suppress the activation of hepatic stem cells (HSCs) and liver fibrosis by downregulating the heat shock protein (HSP) 47/TGF-β/Smad4 signaling pathway [112]. In addition, adipose-derived mesenchymal stem cells (AD-MSCs) significantly reduce elevated levels of liver enzymes and inflammation, such as proinflammatory cytokine levels, in mice with Con A-induced hepatitis [53]. MSCs show promise in treating various forms of acute liver injury, including those caused by lipopolysaccharide (LPS), thioacetamide, ischemia-reperfusion (I/R), and radiation [[113], [114], [115], [116], [117], [118]]. MSC-derived medium has already demonstrated protective effects in these cases [119]. Therefore, the use of MSC-derived exosomes may represent a novel approach to treating various forms of acute liver injury. However, further investigations are necessary to clarify the hepatoprotective effects of MSC-derived exosomes *in vivo*.

### 5.2 Exosomes generated from mesenchymal stem cells and their potential application in the treatment of liver fibrosis and cirrhosis

The utilization of MSCs in animal models of liver fibrosis/cirrhosis has been shown to slow the progression of the disease [54]. Comparable outcomes were also observed when MSC-conditioned media were used, indicating that the therapeutic benefits of MSCs may also be achieved via secreted extracellular vesicles, such as exosomes [54]. Numerous studies have examined the therapeutic benefits of exosomes generated from mesenchymal stem cells in mouse models of liver fibrosis. For example, Li and colleagues discovered that exosomes obtained from human umbilical cord MSCs improved liver fibrosis in carbon tetrachloride-treated Kunming strain mice via their ability to prevent liver cell transformation and decrease collagen production [94]. Research suggests that exosomes primarily work against fibrosis through their contents, particularly miRNAs [120]. miRNAs do not translate into proteins but can regulate gene expression and play essential roles in liver fibrosis, such as controlling the growth, activation, and death of HSCs [121]. A well-known example of a liver-specific miRNA is miR-122, which is involved in liver diseases such as steatohepatitis, viral infections such as HBV and HCV, and liver cancer [[122], [123], [124], [125], [126]]. Some studies have also revealed that miR-122 can improve liver fibrosis by reducing the expression of decapentaplegic protein 4 (Smad4), a primary protein in the TGF-β1 signaling pathway, and limiting collagen production in HSCs [127,128]. Another study revealed that exosomes derived from chorionic plate MSCs, which contain miR-125b, improved liver fibrosis in Sprague‒Dawley rats exposed to carbon tetrachloride by inhibiting the activation of the Hedgehog signaling pathway [95]. Additional data indicated that exosomes from MSCs obtained from adipose tissue, which were modified with miR-122, were more effective at inhibiting the growth and activation of human HSCs by regulating genes related to HSC proliferation and collagen development. These results indicate that altering exosomes with miR-122 might present a promising approach for managing liver fibrosis [129,130]. However, these helpful miRNAs tend to decrease during liver fibrosis, so restoring their normal levels could help improve the patient's condition. Additionally, a new miRNA, miR–181–5p, was identified as playing a critical role in the death of activated HSCs [131]. In a preclinical study of a mouse model of liver fibrosis induced by CCl4, exosomes containing miR–181–5p were shown to increase autophagy and decrease liver fibrosis caused by TGF-β1 by blocking the STAT3/Bcl-2/Beclin 1 pathway in HSCs [132]. A notable decrease in the miR–150–5p level was discovered in HSCs activated during liver fibrosis. However, the administration of miR–150–5p was shown to activate the interferon signaling pathway and induce apoptosis in HSCs [133]. In a mouse model of liver fibrosis caused by CCl4, the expression of C-X-C-motif chemokine ligand 1 (CXCL1) was decreased when adipose MSC-Exos (AdiMSC-Exos) containing miR–150–5p were used, leading to improved liver fibrosis [45].

### 5.3 Exosomes generated from mesenchymal stem cells for the treatment of hepatocellular cancer

Hepatocellular carcinoma (HCC) is a widespread and lethal cancer that is responsible for approximately 90 % of liver cancer cases [134]. Despite advancements in treatments such as surgical resection, liver transplantation, chemoembolization, and immunotherapy, progress in treating HCC has been limited, with unsatisfactory postoperative recovery for patients [135,136]. Primary liver cancer, known as HCC, is characterized by the tumor microenvironment (TME), which enables tumor cells to communicate with nearby and distant cells, and is crucial for the growth and spread of the primary tumor [137]. The TME comprises the extracellular matrix, endothelial cells, cancer-associated fibroblasts, immune cells, and MSCs [138]. Exosomes are essential in the TME and mediate communication that impacts cancer cell survival. Exosomes from MSCs contain proteins and RNAs that exhibit tumor-controlling characteristics, regulating tumorigenesis, angiogenesis, invasion, migration, and drug resistance [139]. MVs derived from BM-MSCs can slow cell cycle progression and trigger apoptosis in HepG2 cells. The injection of MVs into tumors formed in genetically immune-deficient mice significantly reduced tumor growth [128]. According to a recent investigation, exosomes from AD-MSCs in rat N1S1 cells (an orthotopic HCC model) were found to improve the antitumor activity of natural killer T (NKT) cells, reduce the growth of HCC, promote low-grade tumor differentiation, and increase the coefficient of initial evident diffusion, which is a marker of treatment effectiveness [96]. Epithelial‒mesenchymal transition (EMT) occurs when epithelial cells lose their traits and become more phenotypically similar to mesenchymal cells. This promotes angiogenesis linked to cancer and increases cell migration, invasion, and resistance to apoptosis. HUC-MSC-derived exosomal miR-451a may inhibit EMT in HCC cells by targeting metalloprotease 10 (ADAM10) [[140], [141], [142]]. Two additional investigations revealed that exosomes derived from BMSCs, which contain miR-15a and miR-338-3p, inhibited growth, mobility, and cancer cell invasion by reducing the expression of spalt-like transcription factor 4 (SALL4) and E26 transformation-specific-1 (EST1), respectively [[143], [144], [145]]. Exosomes from MSCs are intimately connected to the development, progression, and treatment of HCC. They play a role in immune regulation, HCC invasion, and metastasis, shaping the TME and inducing EMT. Understanding their pursuits in the TME and the potential for EMT could improve HCC treatment [146,147].

## 6 Exosomes derived from mesenchymal stem cells as a drug delivery system for hepatic disorders

Recent advances in biomedical research have identified many possible treatment targets and methods for halting the course of liver disease [148]. On the other hand, many of these targets are unattainable through conventional drugs because they are insoluble, rapidly deactivated, located inside cells, and present in multiple types of cells [149]. While synthetic lipids and nanoparticles have successfully overcome some of these issues as drug vehicles, exosomes, as a natural delivery system, are promising alternatives for delivering drugs to target cells through membrane fusion or endocytosis [150]. Exosomes can be protected from degradation by macrophages or phagocytosis, as they are natural cellular products that can circulate in the body for extended periods [151]. In contrast to liposomes or polymeric nanoparticles, exosomes can bypass the endosomal pathway and avoid lysosomal destruction; thus, they can deliver their payload directly into the cytoplasm, enhancing the effectiveness of transfecting molecules, such as siRNAs [152]. Additionally, exosomes possess other qualities that make them a perfect means of delivering drugs. The contents of proteins and RNAs suggest that they can store a wide range of biological materials [153]. Additionally, they can traverse the challenging blood-brain barrier and are naturally stable due to their composition [[154], [155], [156]]. Furthermore, exosome membranes can be altered to improve the targeting of specific cell types [157,158]. Among the various cell types that produce exosomes, MSCs are the preferred choice for large-scale exosome production for drug delivery. MSC-derived exosomes have been explored as drug delivery vehicles in regenerative medicine and tumor therapy [159,160]. For example, the *in vitro* interaction of BM-MSCs with high concentrations of paclitaxel (PTX) resulted in the packaging and delivery of the drug via exosomes, which showed anti-solid tumor effects against human pancreatic adenocarcinoma [161]. Exosomes derived from BM-MSCs notably decrease the proliferation of glioma xenografts in a Fischer rat model via overexpression of miR-146b or miR-143 and lead to the migration of osteosarcoma cells *in vitro* via delivery of antitumor miRNAs to target tissues. BM-MSCs packed with anti-miR-9 could transfer anti-miR into cocultured glioblastoma cells via the secretion of exosomes, increasing temozolomide sensitivity [75]. In addition, the use of MSC exosome-delivered therapeutic materials in tumor therapy or chemotherapy has also been used in regenerative medicine. For example, exosomes enriched with CXCR4 derived from CXCR4-overexpressing rat bone marrow mesenchymal stem cells have been shown to protect cardiomyocytes against ischemic damage in both a rodent model of myocardial infarction and an *in vitro model*. These beneficial effects were attributed to the upregulation of the Akt signaling pathway, suggesting that CXCR4-enriched exosomes may be a viable therapeutic strategy for promoting cell survival and angiogenesis after myocardial infarction [162]. There are a few studies on the use of exosomes generated from mesenchymal stem cells for pharmacological delivery in hepatic disorders [163,164]. However, the use of MSCs that have explicitly been altered and their conditioned media has improved therapeutic efficacy in liver diseases. Because exosomes contain therapeutic molecules that can be rapidly produced from MSCs, exosome-based drug delivery may offer a promising approach for managing hepatic disorders, including hepatitis, liver fibrosis, and HCC [165]. Furthermore, Bovine milk-derived exosomes have recently gained attention as adaptable and biocompatible nanocarriers for drug delivery, owing to their abundant availability, resilience during gastrointestinal transit, and minimal immunogenicity [166,167]. Besides their transport function, milk exosomes have anti-inflammatory properties due to their bioactive protein and miRNA content [168]. Preclinical studies have shown their capacity to reduce oxidative stress and modulate immune responses in models of inflammation, such as liver injury and metabolic disorders [169,170]. Their favorable oral bioavailability and robust safety profile make them especially appealing for liver-targeted therapies, whether used independently or in conjunction with engineered MSC-derived exosomes within hybrid drug delivery systems.

The effective delivery of MSC-Exos to the targeted liver tissue is essential for improving their therapeutic effectiveness. Native MSC-Exos naturally tend to bind to damaged liver tissue, aided by adhesion molecules, tetraspanins, and integrins on their surface [171,172]. Systemic administration of liver-targeting therapies often results in quick clearance by the mononuclear phagocyte system, which lowers bioavailability [171]. To improve delivery efficiency, several strategies have been suggested: 1. Surface Modification and Targeting: Engineering exosomal membranes with specific ligands can enhance hepatocyte uptake [172]. 2. Hybrid Systems: Combining exosomes with synthetic nanoparticles can boost cargo capacity and extend circulation time [173]. 3. Preconditioning Donor MSCs: Using hypoxia or cytokines can increase the number of beneficial molecules in exosomes for liver targeting [174]. 4. Route of Administration: Local or portal vein delivery can increase hepatic accumulation compared to intravenous methods [175]. Key challenges include preventing opsonization, achieving stability, maintaining Good Manufacturing Practice (GMP)-grade scalability, and ensuring long-term safety. To bring MSC-Exo-based therapies for liver diseases into clinical use, it's crucial to optimize targeting, dosing, delivery routes, and monitoring biodistribution.

## 7 Engineering MSCs and their exosomes for liver regeneration

“Exosome” refers to both naturally available and artificially produced exosomes. Cells, biofluids, and plants are the resources from which natural exosomes are obtained. Most natural exosomes are derived from mammals, and promising future developments are associated with this area of research. On the other hand, engineered exosomes are natural exosomes that have either been subjected to technological modification or have been infused with pharmaceutical compounds [176]. Recent investigations have revealed that nanosized EVs formed from plant cells have features comparable to those of human exosomes. As a result, these nanosized EVs are referred to as “plant-derived exosome-like nanoparticles” (sometimes abbreviated as PDELNs, PLENs, or PDENs) and are classified as EVs [[177], [178], [179]]. Exosomes can be loaded with exogenous therapeutic molecular medications, in addition to the endogenous components already present, to achieve superior benefits against disease. These drugs include proteins, expression vectors, siRNAs, and DNA [180]. MSC-derived exosomes play a crucial role in repairing damaged hepatocytes, including the inactivation of HSCs and the suppression of inflammation. Additionally, they can induce the development of anticancer and chemosensitive responses [181]. Furthermore, when exosomes are engineered to carry a therapeutic agent, they can have a more targeted impact on specific liver complications. Several critical studies on exosome engineering for liver disease treatment are shown in Table 2. Several key obstacles hinder the practical use of exosomes in therapy, including difficulties in isolating and characterizing exosomes, ensuring quality control, replicating functional tests under *both in vitro and in vivo* conditions*,* rapid systemic clearance, an insufficient ability to target, and ambiguous loading efficiency. Therefore, modified exosomes could successfully overcome current restrictions and increase their capacity to load the appropriate therapeutic molecules [182].

**Table 2:** Some applications of engineered exosomes in the treatment of liver diseases.

Liver Disease	MSC-Exosomes Types	Engineering strategy	Model of Disease	Principle of Operation	Impact	References
Liver regeneration	hUCMSC	miR-124 loaded	Partial hepatectomy (70 %) in male 8-week-old Sprague‒Dawley rats	Regulating Foxg1 negatively to promote liver regeneration and reduce liver damage	Accelerate liver rehabilitation from partial resection	(228)
MAFLD	hUCMSC	miR-627-5p loaded	Male rats (6 weeks old and weighing 240–260 g) were fed a high-fat, high-fructose (HFHF) diet for eight weeks.	L-O2 cells' lipid and glucose metabolism were improved by focusing on the FTO gene	MAFLD progression was lessened as hepatocellular injury, lipid accumulation, and glucose and lipid metabolism were all improved *in vivo*	(229)
MAFLD	hUCMSC	Curcumin-Loaded	Healthy male C57BL/6 mice (6 weeks old). These mice were fed a high-fat diet (HFD) for 12 weeks.	Cur-exos from repeated freeze-thawing had better drug retention and higher loading rates than other methods.	Reduced liver damage, inflammation, reactive oxygen species levels, and endoplasmic reticulum stress, while also enhancing lipid metabolism and glucose tolerance	(230)
I/R injury	BMSC	miR-29a-3p	Male Sprague‒Dawley (SD) rats of clean grade	Inhibit ferroptosis by selectively targeting Ireb2	Reduction of steatosis-related liver injury	(231)
I/R injury	BMSC	miR-124-3p	Rats that are specific pathogen-free (SPF)	Reduce the expression of Steap3 to prevent ferroptosis	Reducing graft, I/R damage	(232)
I/R injury	hUCMSC	miR-20a	Male Sprague Dawley rats weighing approximately 300 g	Suppress apoptosis and autophagy mediated by Beclin 1 and FAS	Help heal liver I/R damage	(233)
I/R injury	hUCMSC	miR-1246	Male C57BL/6 mice	Utilize the IL-6-gp130-STAT3 axis, mediated by miR-1246, to alter the ratio of Tregs to Th17 cells.	Improve I/R damage to the liver	(234)
I/R injury	UCMSC	OPDEA-PCL, DSPE-PEG_2000_, or GPEG loaded	Male C57BL/6 mice (6–8 weeks old)	Downregulation of S100A8, S100A9, SELP, and ANXA2, suppression of TLR and MAPK signaling, reduced inflammation and cell death	Improve the stability, liver-targeting ability, and therapeutic efficacy of MSC-derived exosomes against hepatic IRI through surface engineering, while ensuring high biosafety	(235)
HCC	BMSC	NCTD	Male BALB/c nude mice that are four weeks old	Enhance apoptosis, inhibit cell cycle progression, inhibit tumor cell growth, and stimulate cellular uptake.	Manifests potent anticancer activity	(194)
HCC	BMSC	miR-338-3p	Not mentioned	Reducing Expression of EST1	Impede the growth, migration, and invasion of HCC cells and trigger cell death.	(145)
HCC	hUCMSC	miR-451a	Not mentioned	Restrict HCC cell proliferation, migration, invasion, paclitaxel resistance, and epithelial–mesenchymal transformation.	Encourage HCC cells to undergo apoptosis	(236)
HCC	BMSC	miR-127-3p	32 male BALB/c nude mice, each six weeks old and weighing 20–22 g	Control the C5orf66-AS1/miR-127-3p/DUSP1/ERK axis	Stop the malignant behavior of CSCs derived from HCC	(237)
HCC	AMSC	miR-122	Six-week-old male BALB/c nude mice	Negative miR-122 target gene expression control promotes cell death and cell cycle arrest.	Boost the susceptibility of HCC cells to chemotherapy	(238)
Liver fibrosis	MSC	siRNA or ASO	Female BALB/c mice (8 weeks old) with CCl4-induced liver fibrosis	Enhance the effectiveness of STAT3 targeting, reduce STAT3 levels, and reduce ECM accumulation in hepatic fibrosis	Improve liver function significantly	(239)
Liver fibrosis	hUCMSC	BECN1	Mice (BALB/c, female, 4–5 weeks) with CCl4-induced liver fibrosis	Use the xCT/GPX4 pathway to downregulate ferroptosis	Stimulation of target hepatic stellate cells in both *in vitro* and *in vivo*	(240)
Liver fibrosis	MSC	miR-148a	Male C57BL/6 J mouse hepatic fibrosis caused by CCL4 (6–8 weeks)	Deliver miR-148a to macrophages to target the KLF6/STAT3 pathway	Improve the inflammatory response by modifying the *in vivo* phenotypes of macrophages	(241)
Liver fibrosis	AMSC	miR-181-5p	Liver fibrosis in eight-week-old male C57BL/6 mice produced by TGF-1 and CCl4	Block the pathway that leads to STAT3/Bcl2/Beclin 1	Boost autophagy and cut down on fibrosis in the liver	(242)

ALF	AMSC	miR-17	5-6-week-old C57BL/6J mice were given LPS/GalN to cause acute lung injury	Inhibition of TXNIP reduces NLRP3 inflammasome activity	Take on a defending role in ALF	(98)
ALF	hUCMSC	Hepatocyte growth factor (HGF) and glutathione (GSH)-loaded	Carbon tetrachloride-induced acute liver injury, and the Surgical liver injury model	Oxidative Stress Regulation, Anti-Inflammatory Effects, Hepatocyte Regeneration	Improving ALF treatment by the therapeutic, diagnostic, and clinical benefits of the engineered exosomes	(243)
ALF	BMSC	Signal Regulatory Protein Alpha (SIRPα) is expressed	Male mouse ALF models	Dual-mode [1]: SIRPα blocks CD47 on necroptotic hepatocytes, enhancing macrophage-mediated clearance [2]; MSC-EV cargo reprograms macrophages to a pro-regenerative phenotype	Significant reduction in liver injury, enhanced hepatocyte clearance, macrophage reprogramming, improved survival	(244)
Liver injury	hUCMSC	miR-455-3p	Acute liver injury caused by CCl4 in eight-week-old male mice (C57BL/6)	By focusing on the PIK3r1 gene, it prevents the IL-6 signaling pathway from being activated	Reduce excessive monocyte/macrophage activation, reduce liver damage, and enhance systemic homeostasis	(245)

**Abbreviations:** hUCMSC, human umbilical cord mesenchymal stem cell; BMSC, bone marrow mesenchymal stem cell; MAFLD, metabolic-associated fatty liver disease; FTO, fat mass and obesity-associated; HCC, hepatocarcinoma cell; AMSC, adipose-derived mesenchymal stem cell; CSCs, cancer stem cells; MSC, mesenchymal stem cell; ASO, antisense oligonucleotide; CCL4, carbon tetrachloride; I/R, ischemia/reperfusion; OPDEA, poly[2-(N-oxide-N, N-diethylamino)ethyl methacrylate]; GPEG, DSPE-PEG_2000_-galactose.

### 7.1 Mechanisms underlying exosome modification

**Fig. 3:**
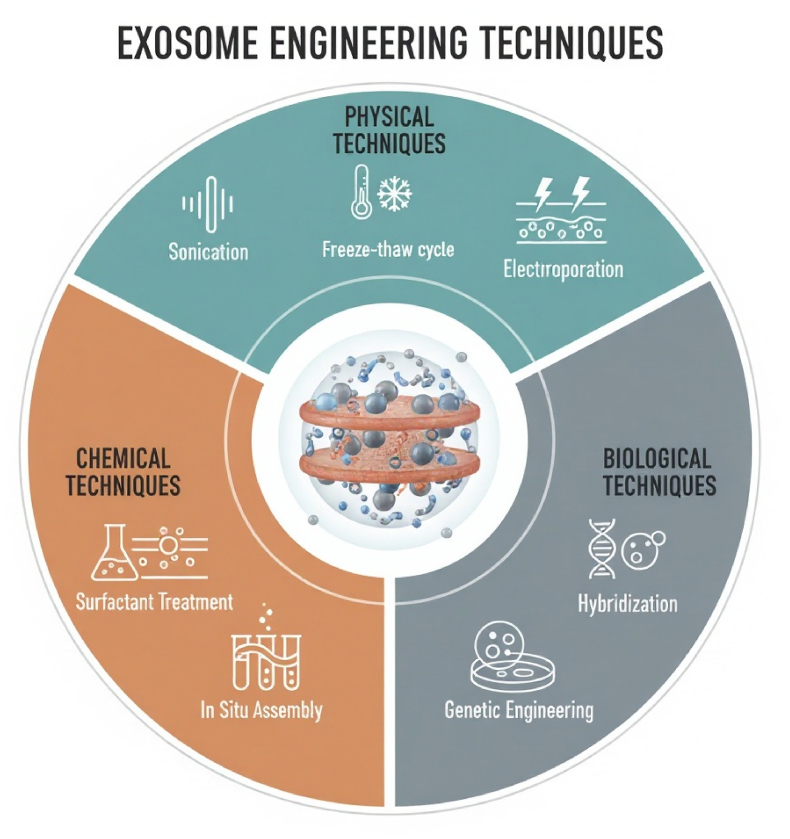
Exosomes separated from MSCs can be engineered and modified *in vitro* via various strategies and utilized for liver disease. Multiple methods for loading cargo into exosomes are categorized into chemical, physical, and biological techniques. Physical techniques, including sonication, freeze-thaw cycles, and electroporation, are used to increase membrane permeability. Chemical methods involve surfactant treatment and in situ assembly for enhanced cargo incorporation. Biological approaches such as preconditioning, genetic engineering, and hybridization with nanovesicles are also employed. These diverse strategies aim to optimize exosome functionality for therapeutic applications.

The engineering of EVs and exosomes involves various strategies, which are primarily categorized into biological, chemical, and physical techniques (see Fig. 3). Furthermore, these strategies can be divided into two main categories: the encapsulation of cargo into exosomes and the alteration of the exosome surface [174]. Encapsulating cargo into exosomes could improve the therapeutic benefits of MSC-derived exosomes. The methods used to package cargo into exosomes can be categorized into two primary groups: endogenous and exogenous cargo loading mechanisms. Endogenous cargo loading methods refer to modifying parental cells via viral vectors and plasmids. On the other hand, exogenous cargo loading involves the direct loading of medications into retrieved MSC-derived exosomes [174]. Endogenous cargo techniques are typically employed for loading endogenous proteins and nucleotides that provide therapeutic properties, whereas exogenous cargo loading techniques are generally utilized for loading small-molecule medicines [183]. Furthermore, Exosome-based hybrid drug delivery systems, a promising platform that combines biological vesicles with synthetic components, are set to transform drug delivery. Lipid-exosome hybrids, typically formed by membrane fusion between exosomes and synthetic lipid nanoparticles, offer higher encapsulation efficiency and stability for large cargoes, such as mRNA and plasmids, while maintaining the intrinsic targeting capabilities of the exosome membrane. This exciting development paves the way for a future of more effective drug delivery [184,185]. Polymer-exosome composites made with biocompatible polymers like polyethylene glycol (PEG), poly lactic-co-glycolic acid (PLGA), or chitosan can enhance circulation time, minimize clearance, and enable stimulus-responsive release [[186], [187], [188]]. These hybrid systems, which combine the advantages of endogenous delivery and engineered nanocarriers, have proven to be highly efficient in the field of regenerative medicine and liver gene therapy. They demonstrate the ability to deliver functional nucleic acids, including luciferase or therapeutic mRNAs, to liver tissue with a significantly higher delivery efficiency compared to natural exosomes alone [184,189]. Hybrid exosome systems, due to their versatility, may represent a next-generation therapeutic toolkit that addresses the size and stability limitations of native vesicles.

#### 7.1.1 Exogenous mechanisms

Typically, physical techniques such as saponin permeabilization, freeze‒thaw cycles, extrusion, or sonication package pharmaceuticals into tailored exosomes. These approaches are used to treat different disorders. For example, sonication was employed to divide exosome membranes into smaller vesicles and introduce medicines through extrusion [190,191]. Electroporation is the predominant technique used to introduce external substances, known as “cargo,” into EVs [192]. This method utilizes an external electric field to create microscopic, reversible openings in phospholipid bilayers. An electric field facilitates the entry of tiny molecules into EVs for drug loading. Electroporation can transport anticancer drugs, such as doxorubicin, into MSC-exosomes, thereby reducing mouse colon cancer tumor development [193]. Liang and colleagues effectively incorporated the exogenous anticancer medication norcantharidin (NCTD) into MSC-exosomes via electroporation. Owing to the inherent liver-targeting characteristics of exosomes, modified exosomes have been used to treat mouse liver cancer models [194]. In addition, incubation, sonication, and dialysis techniques have been employed to attach foreign molecules to MSC-exosomes [174]. In one study, researchers used nanocarriers of exosomes derived from human cord blood MSCs. Genetic engineering was used to modify these exosomes to express an anti-glycan 3 (GPC3) single-chain variable fragment (scFv) on their surface. The adapted exosomes were subsequently loaded with miR-26a mimics via electroporation. These findings indicate that this exosome-based nanocarrier system effectively delivers miR-26a to GPC3-positive HCC cells, efficiently reducing both *in vitro* and *in vivo* tumor growth [195]. In one study, researchers developed engineered EVs that effectively deliver CRISPR/Cas9 components to eliminate the hepatitis B virus (HBV) genome. The engineered EVs were created via protein acylation and heterodimerization by light to increase the loading capacity of the Cas9 protein. Additionally, incorporating vesicular stomatitis virus-glycoprotein (VSV-G) into the EV membrane facilitated the endosomal escape of Cas9 and increased its gene editing activity in recipient cells. Engineered EVs have shown potent antiviral effects by significantly reducing viral antigen and cccDNA levels. They integrated viral DNA in both HBV-replicating and -infected cell models and in HBV-replicating and transgenic mouse models [196].

#### 7.1.2 Endogenous mechanisms

An ever-growing body of documented evidence indicates that the biomolecules that are functionally present in MSC-derived exosomes, including nucleotides and proteins, are crucial for osteogenesis, angiogenesis, immunomodulation, and tissue regeneration across various disease models. Genetic engineering methods, such as viral vectors and plasmids, can selectively manipulate the expression levels of endogenous molecules in MSCs [197,198]. The increasing amount of evidence indicates that MSC-exosomes are crucial for delivering therapeutic benefits to parent cells in diverse disease models through miRNA delivery. Pan et al. demonstrated that MSC-exosomes overexpressing miR-132-3p effectively delivered miRNA to target cells, reducing ROS generation, apoptosis, and tight junction disruption in endothelial cells. Similarly, MSC-exosomes overexpressing miRNA-181a and miR-148a showed more potent therapeutic benefits in treating myocardial and liver ischemia‒reperfusion injuries, respectively [[199], [200], [201]]. Moreover, Lou and colleagues discovered that modifying miR-122 through exosome-mediated communication enhances the effectiveness of MSCs in treating CCl4-induced liver fibrosis by negatively regulating collagen production in HSCs [97]. An alternative is genome editing, wherein parental cells can overexpress the intended cargo, which is then encapsulated in the exosomes [202]. One prominent example of exosome engineering for liver regeneration is the overexpression of miR-122 in AD-MSCs through lentivirus (LV)-mediated transfer of premiR-122 precursor molecules (LV-miR-122). miR-122 can inhibit fibrosis, and its decreased expression is associated with advanced liver diseases such as cirrhosis. The target genes related to HSC activation and proliferation-related miR-122 expression are P4HA1, IGF1R, and CCNG1. In animals with CCl4-induced liver fibrosis, miR-122-containing exosomes decreased the expression of TGF-1 and SMA and reduced HA, P–III–P, ALT, AST, and liver hydroxyproline levels [97,203,204].

### 7.2 Engineering of surfaces

Preloading cargo into exosomes necessitates circumventing the exosome membrane barrier. The surface properties of exosomes are vital for their biodistribution, selective cellular targeting, and therapeutic application. Achieving the desired characteristics is possible through surface engineering techniques. Chemical modification, genetic engineering, or hybrid membrane engineering are viable methods for observing surface changes in exosomes [202,205,206]. In genetic engineering, exosomes can be modified by fusing targeting or ligand molecules with membrane proteins or lipids. This process requires plasmid construction and protein overexpression. Chemical modification techniques can also induce changes in exosomes without disrupting the membrane, but challenges related to membrane complexity and additional purification steps exist [207]. Chemical modifications to alter exosome surfaces can be achieved through noncovalent or covalent interaction strategies. Covalent interactions are generally preferable to noncovalent interactions because they are less susceptible to disruption [207]. During covalent modification, functional groups form strong covalent bonds with exosomes. For example, sulfhydryl groups commonly found on the exosome surface can be targeted as binding sites via the Michael addition reaction between maleimide and sulfhydryl groups. Covalent modification of exosome surfaces involves a crosslinking reaction known as azide‒alkyne cycloaddition or click chemistry, which does not alter the size or function of exosomes [208,209]. The use of toxic chemicals via covalent bonds is considered a disadvantage; therefore, caution should be exercised when this approach is employed in therapeutics [210]. Noncovalent strategies, such as multivalent electrostatic interactions, hydrophobic insertion, and magnetic strength, are typically used to modify biological membranes stably [184,185]. The lipid bilayer of exosomes unequivocally allows for the direct insertion of targeting moieties, thereby enabling the seamless incorporation of functionalized phospholipids into the exosome membrane through straightforward incubation. This method unequivocally facilitates the loading of small lipophilic drugs and is consistently implemented in exosome membrane staining [207]. One study revealed that exosomes altered with cationic pullulan were taken up by HepG2 cells *in vitro*, notably more than unchanged exosomes. This uptake was facilitated by the asialoglycoprotein receptor, which is expressed on HepG2 cells and hepatocytes. When these modified exosomes were injected intravenously into mice with concanavalin A-induced liver injury, they condensed inside the hepatic tissue. This resulted in an enhanced anti-inflammatory stimulus compared to unmodified exosomes. The application of cationized pullulan to modify the surface facilitated the aggregation of exosomes in the liver, thereby improving their therapeutic effect on liver injury [211]. Nakase and colleagues developed a method using cationic lipids and a pH-sensitive fusogenic peptide, called GALA, to enhance the delivery of exosomal cargo. This methodology improves the cytosolic delivery of encapsulated molecules, such as dextran and saporin, into target cells, thereby increasing their biological activity. Optimizing the concentrations of cationic lipids and the GALA peptide is crucial for balancing cellular uptake efficiency and cytotoxicity. Compared with direct delivery, the encapsulation of cargo molecules into exosomes results in increased cellular uptake [212].

### 7.3 Modification of cell culture environments and preconditioning

MSC preconditioning is a valuable strategy for enhancing the therapeutic potential of MSC-derived exosomes, thereby facilitating the development of more effective and targeted exosome-based treatments [213]. Preconditioning involves exposing MSCs to specific stimuli or factors to increase their therapeutic potential via physical, chemical, or biological methods [214]. Typical preconditioning techniques include hypoxia, cytokine, metabolic, and pharmacological preconditioning [215]. In hypoxia preconditioning, MSCs are subjected to low oxygen levels to increase the generation of proangiogenic and anti-inflammatory exosome contents [216]. In cytokine preconditioning, MSCs are treated with specific cytokines (e.g., TGF-β, TNF-α, and IFN-γ) to modify the exosome content [217]. Metabolic preconditioning involves altering nutrient or energy availability to MSCs to modulate exosome composition. Ultimately, pharmacological preconditioning consists of the use of drugs or small molecules to stimulate specific signaling pathways in MSCs, thereby altering the characteristics of the resulting exosomes [218]. Exposing MSCs to low oxygen levels (hypoxia) can increase their growth and genetic stability. It also encourages cells to differentiate into various cell types and increases the expression of genes associated with maintaining stem cell properties. This process may ultimately enhance the effectiveness of MSCs in specific therapeutic applications [174]. Exosomes produced by MSCs in low-oxygen environments have shown great potential for promoting liver regeneration. These exosomes contain elevated levels of growth factors (such as HGF, VEGF, and IGF-1), anti-apoptotic proteins, and pro-angiogenic molecules [174,219]. They have been found to promote the growth of liver cells, prevent liver cell death, and promote angiogenesis in experimental liver injury or disease models [220]. The bioactive contents of these exosomes, including growth factors, miRNAs, and signaling molecules, can activate different signaling pathways in liver cells, such as the PI3K/Akt, MAPK, and Wnt/β-catenin pathways, which are needed for cell proliferation, survival, and blood vessel formation [221]. Exosomes can also deliver their cargo to liver cells, such as hepatocytes and cholangiocytes, to support their regeneration and repair. Research has demonstrated that exosomes produced from low-oxygen MSCs effectively improve liver function, reduce scarring, and accelerate liver regeneration in various types of liver damage, including toxic liver injury, ischemia‒reperfusion injury, and chronic liver disease [221,222]. For example, Xu et al. reported that hypoxic MSCs promote liver regeneration in mice through exosomes. Macrophages assimilate these exosomes, thereby enhancing the polarization of M2 macrophages. They also contain a specific microRNA, miR-182-5p, which inhibits the protein expression of FOXO1 in macrophages, leading to an anti-inflammatory response and demonstrating the capacity of hypoxic exosomes produced by MSCs for liver regeneration [223]. In addition, when MSC secretions are cultured at different oxygen levels (including 21 %, 10 %, 5 %, and 1 %), secretion at 1 % oxygen appears to be the most effective for cell culture. This causes stem cells to release secretions with the maximum ability for liver repair and regeneration [224]. Physiological demands drive the paracrine effectiveness of MSCs and the therapeutic potential of their exosomes. It has been demonstrated that cytokine and inflammatory stimulation enhance the paracrine efficacy of MSCs, controlling the synthesis and excretion of various therapeutic components, including exosomes [225,226]. Zhang et al. demonstrated that pretreatment with exosomes derived from human umbilical cord mesenchymal stem cells (hUCMSC-Exos) has a greater beneficial effect on ALF than does treatment with untreated exosomes. hUCMSC-Exos decreased serum ALT, AST, and proinflammatory cytokine levels, and inhibited the activation of the NLRP3 inflammatory pathway. The anti-inflammatory mechanism is related to the intense expression of microRNA-299-3p in hUCMSC-Exos [227]. Overall, the use of exosomes derived from hypoxic MSCs represents a promising approach for liver regeneration and the treatment of various liver diseases, capitalizing on their enhanced regenerative and proangiogenic properties.

## 8 Future directions and clinical perspectives

Transferring MSC-Exos from the laboratory to the patient's bedside involves overcoming several scientific and regulatory challenges at the same time. During manufacturing, it is crucial to establish strong, GMP-compliant production lines that ensure consistent reproducibility in yield, composition, and therapeutic effectiveness across different batches, without being affected by variability from individual donors [236,237]. Advances in separation technologies, such as tangential flow filtration, size-based chromatography, and microfluidic platforms, are essential for large-scale production of clinical-grade exosomes while preserving their integrity [238]. Engineered exosomes equipped with aptamers, peptides, or antibodies that target liver-specific receptors (e.g., ASGR1, integrins) can enhance site-specific delivery and reduce off-target effects [195,239]. Additionally, hybrid exosomal platforms that combine biological vesicles with synthetic carriers may enable efficient delivery of large molecules like CRISPR/Cas9 components, mRNA, and transcription factors directly into liver cells [240,241]. Immunological safety is also critical, especially regarding long-term biodistribution, clearance kinetics, and potential immune modulation in patients with chronic liver disease [177,237]. Large, placebo-controlled clinical trials should be designed to evaluate various causes, such as viral, alcoholic, and metabolic, focusing not only on short-term biochemical improvements but also on serious outcomes like fibrosis regression, graft-free survival, and overall quality of life [242]. By integrating scalable biomanufacturing, targeted precision, immunological safety, and comprehensive clinical validation, MSC-Exo-based therapies can move from promising preclinical approaches to standard treatments for liver disorders.

## 9 Conclusion

The function of MSC-Exos in the prevention and treatment of acute and chronic liver injury is currently a fascinating subject of investigation, and their therapeutic mechanism has also been actively studied in the context of liver fibrosis, liver cancer, and other diseases. However, large-scale animal studies must confirm its effectiveness in terms of treatment, safety, kinetics, and biodistribution. Additionally, several critical considerations for the clinical use of MSC-Exos in the treatment of liver diseases exist, including the most suitable type of MSCs, the optimal timing for treatment, the optimal dosage regimen, and the preferred route of administration. Furthermore, efficiently producing large amounts of MSC-Exos to meet clinical demands and preserving the stability of MSC-Exos are also pressing issues for the clinical application of MSC-Exos. Researchers have recently generated additional evidence from preclinical experiments, enabling them to develop a framework for the clinical implementation of exosomes and their contents in the treatment of liver diseases. Exosomes are unique carriers for delivering medications due to their ideal features. However, there are significant obstacles to exosome-based therapy, including a short half-life in the circulation, poor targeting, and limited effectiveness, which limit its use. Exosomes with desirable diagnostic and therapeutic properties have been successfully created via exosome engineering and cargo inclusion. The engineering approach, which utilizes homing peptides or specific ligands and component alterations, enhances the therapeutic efficacy of exosomes and facilitates their biodistribution, particularly for liver regeneration and therapy. However, there are still challenges that must be overcome. For example, the range of exosome functions from various sources and the number of exosomes required to achieve the optimal therapeutic impact have not been well investigated. It is necessary to identify relevant cell surface indicators to create highly targeted exosomes for use as efficient drug carriers. The demand for exosomes as biological transporters has risen steadily in recent years, paralleling the growing understanding of these molecules. Improving the detection, diagnosis, and therapeutic strategies for liver illnesses requires further investigation into the efficacy of exosomes in these approaches.

## CRediT authorship contribution statement

**Amir Hossein Kheirkhah:** Writing – original draft, Visualization, Software, Methodology, Investigation, Data curation. **Mohsen Sheykhhasan:** Writing – review & editing, Validation, Conceptualization. **Faezeh Hosseinzadeh:** Writing – review & editing, Methodology, Investigation, Conceptualization. **Leyla Fath-Bayati:** Writing – review & editing, Supervision, Project administration, Methodology, Investigation, Formal analysis, Data curation.

## Consent to participate

Not applicable.

## Ethics approval

Not applicable.

## Consent for publication

Not applicable.

## Funding

This study is part of a thesis supported by Qom University of Medical Sciences (Grant Number: IR.MUQ.AEC.1402.030).

## Declaration of competing interest

The authors declare that they have no known competing financial interests or personal relationships that could have appeared to influence the work reported in this paper.

## Data Availability

This study is a literature review and does not include any original data or code. All information analyzed and discussed in this article is already publicly available in the cited references.
